# Gender-Specific Determinants of Frailty in Aging People with HIV: Evidence for a Multidimensional Vulnerability Phenotype in Women

**DOI:** 10.3390/v18070742

**Published:** 2026-07-04

**Authors:** Patricia Echeverría, Jordi Puig, Ana Martínez, Itziar Arrieta, Isabel Arnau, Lucía Bailón, Carla Estany, Begoña Lemos, Anna Bonjoch, Robert Güerri, Eugenia Negredo

**Affiliations:** 1 Infectious Diseases Service, Hospital Universitari Germans Trias i Pujol, 08916 Badalona, Spain; jpuig@scienhub.org (J.P.); lbailon@lluita.org (L.B.); blemos@lluita.org (B.L.); abonjoch@lluita.org (A.B.); enegredo@lluita.org (E.N.); 2 Faculty of Medicine, Universitat Autònoma de Barcelona, 08193 Bellaterra, Spain; 3 Fundació Lluita contra les Infeccions, 08916 Badalona, Spain; amartinez@scienhub.org (A.M.); cestany@lluita.org (C.E.); 4 Hospital del Mar, 08003 Barcelona, Spain; iarrieta@hmar.cat (I.A.); iarnau@hmar.cat (I.A.); rguerri@hmar.cat (R.G.); 5 Centro de Investigación Biomédica en Red (CIBERINFEC), 28029 Madrid, Spain; 6 Faculty of Medicine, University of Vic-Central University of Catalonia (UVic-UCC), 08500 Vic, Spain

**Keywords:** gender differences, aging, frailty phenotype, multidimensional vulnerability, women aging with HIV, gender-sensitive HIV

## Abstract

**Background:** Gender differences in aging among people with HIV (PWH) remain poorly characterized. Women with HIV (WWH) may experience more complex aging trajectories, due to the interplay of biological, clinical, and psychosocial factors. In this context, we aimed to investigate gender-specific determinants of frailty among older people with HIV, with a particular focus on women, to better inform tailored clinical care. **Methods:** Cross-sectional analysis of the Over50 Cohort, including PWH aged ≥50 years from two tertiary hospitals in Spain. Participants underwent a comprehensive geriatric assessment across demographic, clinical, functional, cognitive, psychological, and social domains. Gender-stratified multivariable analyses examined frailty (by Fried criteria) and associated factors. **Results:** Among 588 participants, 139 (23.6%) were cisgender WWH. Despite younger age and better immune status, WWH showed higher prevalence of frailty (17% vs. 9%), musculoskeletal disease (47% vs. 28%), depressive symptoms (45% vs. 30%), sleep disturbances (10% vs. 5%), and cognitive complaints (23% vs. 11%). Men with HIV (MWH) more frequently had cardiovascular (48% vs. 35%) and renal disease (22% vs. 15%). In multivariable models, frailty in WWH was independently associated with musculoskeletal disease (OR 3.85), cognitive impairment (OR 3.21), depressive symptoms (OR 2.67), and malnutrition (OR 2.14). In MWH, frailty was associated with musculoskeletal disease, cognitive impairment, malnutrition, and older age. **Conclusions:** Frailty exhibits gender-specific patterns: a multidimensional phenotype in WWH versus age-driven in MWH, supporting tailored, gender-responsive care integrating geriatric, mental, and musculoskeletal health.

## 1. Introduction

According to UNAIDS, an estimated 40.9 million people were living with HIV globally in 2025, reflecting the continuing scale of the epidemic and the success of antiretroviral therapy in extending survival, with women and girls accounting for 51% of all people living with HIV and approximately 41% of new HIV diagnoses in the EU/EEA, a distribution that has remained relatively stable over the past decade [1].

Although HIV infection affects men and women through similar biological mechanisms, substantial sex-related differences have been described, across biological, clinical, and psychosocial domains.

Overall, women with HIV (WWH) exhibit a higher burden of aging-related comorbidities such as osteoporosis, chronic lung disease, and diabetes, whereas men with HIV (MWH) more frequently present cardiovascular disease, dyslipidemia, and liver disease [2,3].

Frailty, a geriatric syndrome characterized by reduced physiological reserve and increased vulnerability to stressors, has emerged as a key determinant of adverse outcomes in aging PWH, including disability, multimorbidity, and mortality [4,5,6,7,8]. Recent multicenter and international cohort studies report frailty prevalences ranging from 10% to 22% among older PWH, with WWH consistently showing greater vulnerability compared with MWH [9,10,11].

Similarly, psychiatric disorders, particularly depression and anxiety, remain highly prevalent among PWH and disproportionately affect WWH, contributing to poorer quality of life, functional decline, and worse clinical outcomes [12]. In addition, social determinants such as poverty, stigma, and gender-based violence further exacerbate health disparities among WWH, influencing mental health, treatment adherence, and access to care [13,14].

Furthermore, the menopausal transition represents a critical and often under recognized factor in aging WWH, with hormonal changes potentially accelerating trajectories of frailty, musculoskeletal decline, neurocognitive impairment, mood disorders and sexual dysfunction [15,16]. Sexual dysfunction has also emerged as a frequently neglected but clinically relevant issue in aging PWH, with WWH reporting higher rates of hypoactive sexual desire and dyspareunia, while erectile dysfunction predominates among MWH [16].

These gender-based differences highlight the need for gender-sensitive approaches to HIV care. This study examined gender-based disparities, comparing comorbidities, geriatric syndromes, cognitive impairment, and psychosocial factors within the Over50 Cohort, a prospective cohort of PWH ≥ 50 years in Barcelona, Catalonia, and Spain [17,18]. We also aimed to characterize aging-related health trajectories in PWH, with a specific focus on frailty, to inform gender-tailored clinical strategies and optimize long-term care.

## 2. Materials and Methods

### 2.1. Study Design and Population Section

This study is a cross-sectional analysis of data derived from the Over50 Cohort using the most recent available data at a fixed cut-off in January 2026. Over50 Cohort is a prospective cohort of individuals aged ≥50 years with chronic HIV infection who are consecutively enrolled during routine visits to the HIV units of two tertiary care hospitals in Barcelona, Spain. Enrollment began in September 2015 and is ongoing. All participants were cisgender (sex assigned at birth concordant with gender identity) according to available clinical records, no transgender or gender-diverse individuals were included in this analysis. Epidemiological and clinical data were obtained from electronic medical records and a comprehensive geriatric assessment encompassing medical, psychological, functional, and social domains is included [17,18]. For the present analysis, data from the most recent available assessment were used. A total of 588 PWH were included in the current analysis.

The Cohort Over50 was approved by the Research Ethics Committees and health authorities of Germans Trias i Pujol University Hospital (Spain) on 9 October 2015 (approval number PI-15-106), and Hospital del Mar (Spain) on 23 March 2022 (approval number PI-16-068). The study was conducted in accordance with the Declaration of Helsinki and the principles of Good Clinical Practice (GCP). Participation was voluntary, free, and independent; all participants provided written informed consent prior to study participation, and all data were anonymized to ensure participant confidentiality.

### 2.2. Study Objectives and Endpoints

We aimed to characterize gender-related differences in aging-associated conditions among people with HIV (PWH) aged ≥50 years using a hierarchical analytical approach. First, we performed a descriptive comparison of clinical, geriatric, and psychosocial domains, including cognitive, nutritional, and functional status; all multidimensional domains were assessed using validated instruments, with detailed definitions available in Table 1 and the referenced methodological publications [17,18]. Second, we identified gender-specific frailty patterns based on the Fried frailty phenotype criteria; and third, we examined the independent associations between frailty and multidimensional determinants through stratified multivariable analyses.

### 2.3. Statistical Analysis

Statistical analyses were performed to describe the cohort and evaluate gender-related differences in aging-associated conditions among PWH aged ≥50 years. Categorical variables were summarized as frequencies (percentages) and compared using the χ^2^ or Fisher’s exact test, as appropriate, whereas continuous variables were described as medians (IQR) and compared using the Mann–Whitney U test.

Frailty, defined according to the Fried criteria, was analyzed as a binary outcome.

Descriptive analyses were performed using Jamovi (version 2.7.13).

Multivariable logistic regression models were performed using the statsmodels package, whereas pandas (v.3.0.4), NumPy (v. 2.5.0), and SciPy (v. 1.18.0) were used for data management and supplementary statistical analyses. Effect sizes are presented as adjusted odds ratios (aORs) with 95% confidence intervals (CIs). All statistical tests were two-sided, and statistical significance was defined as *p* < 0.05.

Missing data were primarily observed among participants enrolled early in the cohort, before certain assessments were systematically incorporated into the standardized protocol, resulting in time-dependent missingness consistent with a missing-at-random mechanism. Variable-specific denominators (n/N) were therefore used for all analyses.

## 3. Results

### 3.1. Demographic and HIV-Related Characteristics

A total of 588 PWH aged ≥50 years were included in the analysis, of whom 139 (23.6%) were cisgender WWH and 449 (76.4%) were cisgender MWH.

Table 1 summarizes participants’ epidemiological characteristics, comorbidity burden, functional and geriatric assessments, including cognitive, psychosocial, and quality of life domains.

The median age was comparable between groups (65 vs. 67 years, *p* = 0.09), as well as the proportion of individuals aged ≥70 years (21% vs. 30%, *p* = 0.05).

WWH had higher median CD4+ counts than MWH (711 vs. 665 count cel/mm^3^, *p* < 0.01), while no significant gender differences were observed in the prevalence of severe immunosuppression (CD4+ ≤200 count cells/mm^3^) (4% vs. 2%, *p* = 0.25). Both groups demonstrated high rates of viral suppression, and the median duration of antiretroviral therapy was similar between groups (27 vs. 23 years, *p* = 0.89) (Table 1).

### 3.2. Comorbidities, Geriatric Syndromes and Psychosocial Domains

The overall burden of comorbidities was comparable in both genders; however, percentage of PWH with ≥3 comorbidities was lower in WWH than in MWH (52% vs. 65%; OR 0.58, 95% CI 0.39–0.86, *p* < 0.01).

Gender-stratified analyses revealed distinct patterns across clinical, functional, geriatric, neurocognitive, and psychosocial domains (Table 1). To note, WWH showed significantly higher odds of musculoskeletal comorbidity compared with MWH (47% vs. 28%; OR 2.31, 95% CI 1.55–3.44, *p* < 0.01) while cardiovascular comorbidities were more prevalent in MWH (48% vs. 35%; OR 0.58, 95% CI 0.39–0.85, *p* < 0.01).

Functional impairment [Short Physical Performance Battery impaired (SPPB), Barthel, and Lawton & Brody] and sensory deficits (hearing, vision) showed no meaningful gender-based differences. In contrast, several geriatric syndromes showed higher prevalence in WWH than in MWH. Frailty was more common in WWH (17% vs. 9%; OR 2.08, 95% CI 1.20–3.59, *p* < 0.01), as were sleep disorders (10% vs. 5%; OR 2.14, 95% CI 1.05–4.35, *p* = 0.04); and altered nutrition (37% vs. 28%; OR 1.49, 95% CI 1.01–2.21, *p* = 0.05) showed a borderline trend towards higher prevalence in WWH.

Sexual dysfunction was similarly high in both sexes but slightly more common in WWH (66% vs. 56%, OR 1.52, 95% CI 0.99–2.33, *p* = 0.06), without reaching statistical significance. However, when examining participants with any sexual dysfunction, the type of dysfunction differed significantly by sex: no sexual activity was more common among WWH compared with MWH (39% vs. 16%, *p* < 0.01), whereas erectile dysfunction was very prevalent in MWH, representing 47% of sexual dysfunction cases, and vaginal problems accounted for a meaningful proportion of dysfunction types in WWH (20% overall).

In the neurocognitive domain, WWH exhibited a twofold higher prevalence of cognitive complaints (23% vs. 11%; OR 2.33, 95% CI 1.38–3.93, *p* < 0.01) and significantly higher rates of depressive symptoms (45% vs. 30%; OR 1.88, 95% CI 1.25–2.82, *p* < 0.01).

Quality of life and social support measures were similar across genders; even so, low quality of life (19% vs. 13%, OR 1.51, 95% CI 0.81–2.80, *p* = 0.25) and mild/moderate deteriorate recourse (20% vs. 13%, OR 1.68, 95% CI 0.63–4.47, *p* = 0.50) were more common in WWH than MWH, but without reaching statistical significance.

These findings are summarized in Table 1, which presents odds ratios and 95% confidence intervals alongside gender-specific absolute risks.

### 3.3. Comparative Analysis Between Frail and Robust Participants in Overall Population

In the comparative analysis between frail and robust participants, frailty was consistently associated with a higher burden of adverse conditions across demographic, clinical, functional, geriatric, neurocognitive, and social domains (Table 2).

Regarding demographic characteristics, individuals aged 70 years or older showed significantly higher odds of frailty compared with robust participants (26% vs. 11%; OR 2.86, 95% CI 1.40–5.84, *p* < 0.01). Female gender was also associated with frailty (36% vs. 21%; OR 2.14, 95% CI 1.17–3.92, *p* = 0.01).

Multimorbidity, defined as the presence of three or more chronic conditions, was strongly associated with frailty (82% vs. 48%; OR 4.82, 95% CI 2.47–9.41, *p* < 0.01). Furthermore, frail participants exhibited significantly increased odds of several specific comorbidity categories, including musculoskeletal disease (58% vs. 30%; OR 3.13, 95% CI 1.73–5.67, *p* < 0.01), neurological disease (27% vs. 8%; OR 4.47, 95% CI 2.10–9.53, *p* < 0.01), psychiatric disorders (47% vs. 17%; OR 4.48, 95% CI 2.4–8.31, *p* < 0.01), respiratory disease (33% vs. 12%; OR 3.58, 95% CI 1.76–7.2; *p* < 0.01), and neoplastic disease (24% vs. 9%; OR 3.18, 95% CI 1.49–6.78, *p* < 0.01).

Measures of functional status clearly distinguished frail from robust participants. Frail individuals were significantly more likely to exhibit SPPB impairment (47% vs. 3%; OR 30, 95% CI 3.95–229; *p* < 0.01), and limitations in instrumental activities of daily living assessed by the Lawton Index (24% vs. 0%; OR 27.7, 95% CI 1.66–464, *p* < 0.01), whereas no significant differences were observed in basic activities of daily living measured by the Barthel Index.

Multiple geriatric syndromes were also strongly associated with frailty. Frail participants had markedly increased odds of sleep disturbance (24% vs. 2%; OR 15.2, 95% CI 4.87–47.48, *p* < 0.01) and malnutrition risk (52% vs. 19%; OR 4.5, 95% CI 2.47–8.22, *p* < 0.01). Sensory impairments were common, with strong associations observed for visual impairment (46% vs. 8%; OR 9.27, 95% CI 4.58–18.76, *p* < 0.01) and hearing impairment (55% vs. 29%; OR 2.89, 95% CI 1.63–5.15, *p* < 0.01). Sexual dysfunction was also significantly more prevalent among frail participants (80% vs. 51%; OR 3.97, 95% CI 2.01–7.87, *p* < 0.01).

Neurocognitive and mental health variables exhibited some of the most robust associations with frailty. Both subjective cognitive complaints (41% vs. 7%; OR 9.64, 95% CI 4.57–20.34, *p* < 0.01) and objectively measured cognitive impairment (47% vs. 9%; OR 9.30, 95% CI 4.46–19.36, *p* < 0.01) were strongly associated with frailty. Depressive symptoms were also significantly more frequent among frail participants (69% vs. 24%; OR 6.84, 95% CI 3.66–12.80, *p* < 0.01), highlighting the substantial contribution of neuropsychiatric vulnerability to the frailty phenotype.

Quality of life was markedly reduced among frail participants, with low quality of life strongly associated with frailty status (42% vs. 7%; OR 8.99, 95% CI 4.12–19.61, *p* < 0.01). Finally, social resource indicators suggested increased social vulnerability among frail individuals: higher odds of mild (29% vs. 9%; OR 4.1, 95% CI 1.01–16.57.1, *p* = 0.06) and severe social deterioration (10% vs. 0%; OR 11.7, 95% CI 0.53–254.4, *p* = 0.09) were observed among frail people.

### 3.4. Patterns of Frailty According to Gender

Among frail PWH, gender-based differences were explored across demographic and psychosocial characteristics, comorbidity burden, functional status, and geriatric syndromes (Table 3).

Despite broad similarity across many domains, a distinctive gender specific frailty pattern emerged. Frail WWH showed a substantially higher prevalence of musculoskeletal comorbidities compared with MWH (83% vs. 41%; OR 6.88, 95% CI 2.04–23.2, *p* < 0.01) as well as markedly greater burden of objective cognitive impairment (63% vs. 29%; OR = 4.03, 95% CI: 1.4–12.0, *p* = 0.01), suggesting a musculoskeletal–neuropsychiatric vulnerability profile in WWH. Several additional domains, although not statistically significant, displayed consistent and clinically meaningful directional differences. Trends toward higher odds among WWH were noted for psychiatric disorders (63% vs. 39%; OR 2.69, 95% CI 0.92–7.85, *p* = 0.07). Social deterioration also was more frequent and more severe in WWH, with higher proportions of both mild–moderate deterioration (14% vs. 8%; OR 2, 95% CI 0.11–38, *p* = 1.00) and severe deterioration (43% vs. 23%; OR 3, 95% CI 0.35–18, *p* = 0.61).

In contrast, frail MWH only showed a higher prevalence of visual impairment compared with WWH (28% vs. 51%; OR 0.38, 95% CI 0.15–0.95, *p* = 0.02).

### 3.5. Associations with Frailty According to Gender

In gender-stratified multivariable analyses, several factors were independently associated with frailty, with notable sex-specific patterns (Table 4).

Among WWH, frailty was strongly associated with musculoskeletal disease (aOR 3.85, 95% CI 1.72–8.61; *p* < 0.01), cognitive impairment (aOR 3.21, 95% CI 1.45–7.10; *p* < 0.01), depressive symptoms (aOR 2.67, 95% CI 1.28–5.54; *p* < 0.01), and malnutrition (aOR 2.14, 95% CI 1.02–4.51; *p* = 0.04). Age ≥ 70 years showed a non-significant trend (aOR 1.72; *p* = 0.09).

In MWH, frailty was independently associated with older age (aOR 2.94, 95% CI 1.85–4.68; *p* < 0.01), musculoskeletal disease (aOR 2.21, 95% CI 1.34–3.66; *p* < 0.01), cognitive impairment (aOR 2.54, 95% CI 1.48–4.35; *p* < 0.01), and malnutrition (aOR 2.36, 95% CI 1.33–4.18; *p* < 0.01). Depressive symptoms and sleep disturbances were not significantly associated.

Significant interactions by gender were observed for age (*p*-interaction = 0.03) and depressive symptoms (*p*-interaction = 0.04), indicating stronger associations in MWH for age and in WWH for depression.

## 4. Discussion

In this well-characterized European cohort of PWH aged ≥50 years, we identified clinically meaningful sex differences across geriatric, neurocognitive, and psychosocial domains. Perhaps the most striking finding was that chronological age, a cornerstone of conventional frailty risk assessment, remained independently associated with frailty in MWH but not in WWH. This observation suggests that frailty in WWH may not be adequately captured by age-based screening strategies alone and instead reflects the cumulative impact of multidimensional vulnerabilities. Consistent with this hypothesis, WWH exhibited a distinct frailty phenotype characterized by the coexistence of musculoskeletal disease, cognitive impairment, depressive symptoms, and nutritional vulnerability, supporting the concept of a neuropsychiatric–musculoskeletal pattern of frailty that differs fundamentally from the more age-driven trajectory observed in men.

Notably, WWH showed a higher prevalence of frailty and several aging-related conditions despite more preserved immune status and a lower burden of multimorbidity than MWH, in agreement with previous studies [3,4,5,6,7,10,14,15,16,19]. Crucially, our findings extend beyond describing prevalence differences by identifying the specific factors underlying frailty in women. The convergence of musculoskeletal, cognitive, nutritional, and psychosocial vulnerabilities suggests that frailty in WWH emerges through interacting multidomain processes rather than as a simple consequence of chronological aging.

Frailty in WWH was driven by the convergence of musculoskeletal impairment, cognitive dysfunction, depressive symptoms, sleep disorders and malnutrition, whereas frailty in MWH is more strongly associated with chronological aging, alongside physical and cognitive decline, reflecting a more traditional aging-related phenotype. These data reinforce the concept that frailty is a multidimensional construct integrating biological, cognitive, and psychosocial domains [6,7,8,9,10,11,12,20], but further demonstrate that its determinants are strongly gender specific. The identification of a musculoskeletal–neurocognitive–psychosocial vulnerability cluster in our WWH represents a novel contribution. Previous cohorts (WIHS, MACS) showed higher frailty in women but did not characterize drivers [19,20,21].

Our multivariable analysis shows that in WWH, frailty is independently associated with multidomain impairment, with a strong psychological component (depression, sleep disturbances), whereas age loses significance. By contrast, MWH exhibits a predominantly age-driven frailty trajectory, consistent with patterns observed in the general population and prior HIV cohorts. Together, these findings support a syndemic framework in which interacting biological processes (such as menopause-related hormonal changes and sarcopenia) and structural determinants (including psychosocial vulnerability and gender-related inequities) converge to shape frailty trajectories in women aging with HIV [7,8,9,10,11,12,13,14,16,20,21,22,23], alongside cumulative exposure to gendered social adversity, stigma, and inequitable access to care.

These findings align with emerging evidence from the WIHS Cohort and other studies highlighting the role of depression and cognitive impairment in frailty among WWH [12,14,15,19,20,21], and with recent frameworks positioning sex and gender as central determinants of biological aging [23]. Together, they support a paradigm shift in which frailty in WWH is understood as a life-course-mediated, multidimensional vulnerability state, rather than a simple function of aging.

In parallel, the higher prevalence of cardiovascular disease among MWH is consistent with the established literature and likely reflects differential exposure to cardiometabolic risk factors and health-related behaviors, including tobacco use, alcohol consumption, and substance use [5,24,25]. These gender-specific patterns emphasize the need to interpret aging-related comorbidity through both biological and behavioral lenses.

The clinical implications are immediate. Early identification of frailty is critical, given its strong association with disability, poor quality of life, and mortality in PWH [7,8,26]. In WWH, the clustering of vulnerabilities supports the need for integrated, gender-responsive care models that incorporate mental health and cognitive screening, as well as menopause-informed management [22,23,27,28].

Although menopause likely represents a pivotal driver of aging trajectories in women with HIV, the absence of data on hormone replacement therapy precludes assessment of its potential modifying role. In MWH, targeted prevention strategies should prioritize optimization of modifiable risk factors, particularly cardiometabolic health and lifestyle behaviors.

Sexual dysfunction, highly prevalent yet under-recognized in both sexes, remains an unmet clinical need. Together, these results support the concept that frailty in people aging with HIV arises through gender-divergent pathways, reflecting the interplay between biological mechanisms such as menopause, vascular disease, and metabolic alterations, and gender-related determinants including stigma, relational dynamics, and social vulnerability [13,14,19,22,25,27,28,29]. Its systematic assessment should be incorporated into routine HIV care.

Despite a greater objective burden of vulnerability, quality of life was comparable between WWH and MWH, suggesting the presence of adaptive coping strategies or resilience. This discordance between objective functional vulnerability and subjective health perception may indicate that important protective factors remain uncaptured by conventional clinical assessments and warrants further investigation.

This study has several limitations. Its cross-sectional design precludes causal inference and limited assessment of the potential impact of the treatment era, cumulative antiretroviral exposure, and historical exposure to older antiretroviral regiments. Selection bias might have resulted in underrepresentation of the most vulnerable individuals. Findings are restricted to cisgender populations and may not be generalizable to transgender individuals. Menopausal status was not systematically recorded despite inclusion of women aged ≥50 years, precluding evaluation of its potential contribution to frailty. Although sexual dysfunction was highly prevalent, the absence of significant sex differences limited its relevance as a sex-specific determinant. Finally, some variables were self-reported and stratified analyses were constrained by sample size. Nevertheless, the use of a standardized multidimensional assessment in a large, well-characterized cohort strengthens the robustness and clinical relevance of our findings.

## 5. Conclusions

Aging with HIV is characterized by gender-specific vulnerability pathways. Beyond descriptive differences, our findings provide evidence that frailty operates through gender-divergent pathways, with distinct underlying determinants rather than a uniform aging process. WWH experience a distinct, multidimensional frailty phenotype, driven by musculoskeletal, neurocognitive, and psychosocial factors largely independent of chronological age, whereas frailty in MWH follows a more conventional age-related trajectory. The lack of an independent association between age and frailty in WWH suggests that clinically relevant vulnerability may not be adequately captured by chronological age alone, highlighting the need for gender-responsive and multidimensional screening approaches. Longitudinal research is needed to elucidate underlying mechanisms and to inform targeted interventions aimed at improving healthspan in aging PWH.

## Figures and Tables

**Table 1:** Gender-based differences in categorical clinical variables among people with HIV aged ≥50 years.

Variable	WWH (n = 139)	MWH n = 449	OR (95% CI)	*p*-Value
Demographics				
Age, median (IQR)	65 (50, 90)	67 (50, 93)	-	0.09
≥60 years, n/N (%)	87 (64)	315 (71)	0.71 (0.48–1.06)	0.09
≥70 years, n/N (%)	29 (21)	134 (30)	0.62 (0.39–0.98)	0.05
HIV-Related data				
CD4+ count cel/mm^3^, median (IQR)	711 (118, 2026)	665 (87, 2168)	-	<0.01
CD4+ ≤200 count cells/mm^3^, n (%)	4 (4)	6 (2)	2.19 (0.61–7.87)	0.25
Suppressed viral load (<50 copies/mL), %	100	100	-	1.00
Time on antiretroviral therapy, median (IQR)	27 (20, 32)	23 (16, 29)	-	0.89
Comorbidity burden				
Number of comorbidities, median (IQR)	3 (0, 12)	3 (0, 16)	-	0.98
≥3 comorbidities, n/N (%)	72/139 (52)	292/449 (65)	0.58 (0.39–0.86)	<0.01
Comorbidity domains				
Endocrine, n/N (%)	80/139 (58)	250/449 (56)	1.09 (0.73–1.62)	0.77
Cardiovascular, n/N (%)	49/139 (35)	215/449 (48)	0.58 (0.39–0.85)	<0.01
Renal, n/N (%)	20/139 (15)	100/449 (22)	0.59 (0.35–1.00)	0.06
Musculoskeletal, n/N (%)	65/139 (47)	126/449 (28)	2.31 (1.55–3.44)	<0.01
Neurological, n/N (%)	12/139 (10)	55/449 (12)	0.80 (0.41–1.55)	0.30
Respiratory, n/N (%)	20/139 (14)	65/449 (14)	1.00 (0.58–1.71)	1.00
Psychiatric, n/N (%)	34/139 (24)	98/449 (22)	1.16 (0.73–1.84)	0.59
Neoplasm, n/N (%)	23/139 (17)	54/449 (12)	1.45 (0.83–2.53)	0.21
Gastrointestinal, n/N (%)	12/139 (9)	56/449 (12)	0.73 (0.38–1.39)	0.27
Hepatic, n/N (%)	27/139 (19)	78/449 (17)	1.15 (0.70–1.86)	0.67
Others, n/N (%)	37/139 (27)	113/449 (25)	1.10 (0.70–1.72)	0.81
Functional status				
Short Physical Performance Battery (SPPB) impaired, n/N (%)	35/138 (25)	86/414 (21)	1.27 (0.80–2.00)	0.31
Barthel index impaired, n/N (%)	1/139 (1)	3/440 (2)	0.99 (0.10–9.85)	1.00
Lawton & Brody index impaired, n/N (%)	59/139 (42)	161/440 (37)	1.24 (0.84–1.83)	0.25
Geriatric syndromes				
Frailty (by Fried criteria), n/N (%)	24/139 (17)	41/449 (9)	2.08 (1.20–3.59)	<0.01
Sleep disturbance (by Pittsburg Sleep Quality Index), n/N (%)	14/139 (10)	22/443 (5)	2.14 (1.05–4.35)	0.04
Malnutrition risk (by MNA), n/N (%)	52/139 (37)	127/449 (28)	1.49 (1.01–2.21)	0.05
Visual impairment, n/N (%)	27/135 (20)	77/418 (18)	1.15 (0.69–1.90)	0.77
Hearing impairment (by Hearing difficulties detection), n/N (%)	46/136 (34)	158/436 (36)	0.91 (0.61–1.37)	0.68
Neurocognitive & Mental Health				
Cognitive complaint (by EACS cognitive screening questions), n/N (%)	32/139 (23)	51/449 (11)	2.33 (1.38–3.93)	<0.01
Cognitive performance impairment (by NEU Screening), n/N (%)	22/82 (27)	83/271 (31)	0.82 (0.48–1.39)	0.60
Depressive symptoms (by Geriatric Depression Scale-Short Form GDS-15), n/N (%)	57/128 (45)	126/416 (30)	1.88 (1.25–2.82)	<0.01
Quality of life (by a self-developed instrument) & Social support (by OARS)				
Low quality of life (QoL), n/N (%)	17/91 (19)	39/296 (13)	1.51 (0.81–2.80)	0.25
Good social support, n/N (%)	27/35 (77)	87/104 (83)	0.70 (0.29–1.68)	0.54
Mild deterioration, n/N (%)	7/35 (20)	14/104 (13)	1.68 (0.63–4.47)	0.50
Severe deterioration, n/N (%)	1/35 (3)	3/104 (3)	0.99 (0.10–9.85)	1.00

Data are presented as median (interquartile range [IQR]) or number and proportion (%). Categorical variables were compared using Chi-square or Fisher’s exact test; continuous variables were analyze using the Mann–Whitney U test. Statistical significance was set at *p* < 0.05. Odds ratios (OR) with 95% confidence intervals (CI) were calculated using exact methods. Tests performed using 2 × 2 tables (presence vs. absence) by gender, *p*-values indicate statistical significance at alpha = 0.05 without adjustment for multiple comparisons. Frailty was defined using Fried criteria.

**Table 2:** Comparison of clinical, comorbidity, functional, and neurocognitive domains between frail and robust adults living with HIV.

Variable	Frail Adults n = 66	Robust Adults n = 194	OR (95% CI)	*p*-Value
Demographics				
Female, n/N (%)	24/66 (36)	41/194 (21)	2.14 (1.17–3.92)	0.01
Male, n/N (%)	42/66 (64)	153/194 (79)	0.47 (0.26–0.86)	0.02
≥60 years, n/N (%)	45/66 (68)	122/194 (63)	1.26 (0.70–2.29)	0.46
≥70 years, n/N (%)	17/66 (26)	21/194 (11)	2.86 (1.40–5.84)	<0.01
HIV-Related data				
CD4+ ≤200 count cells/mm^3^, n (%)	3/66 (5)	2/194 (1)	4.44 (0.71–26.6)	0.10
Comorbidity burden				
≥3 comorbidities, n/N (%)	54/66 (82)	93/194 (48)	4.82 (2.47–9.41)	<0.01
Comorbidity domains				
Endocrine, n/N (%)	40/66 (61)	94/194 (49)	1.64 (0.93–2.89)	0.11
Cardiovascular, n/N (%)	31/66 (47)	72/194 (37)	1.50 (0.85–2.64)	0.19
Renal, n/N (%)	18/66 (27)	41/194 (21)	1.40 (0.74–2.66)	0.31
Musculoskeletal, n/N (%)	38/66 (58)	59/194 (30)	3.13 (1.73–5.67)	<0.01
Neurological, n/N (%)	18/66 (27)	15/194 (8)	4.47 (2.10–9.53)	<0.01
Respiratory, n/N (%)	22/66 (33)	23/194 (12)	3.58 (1.76–7.2)	<0.01
Psychiatric, n/N (%)	31/66 (47)	32/194 (17)	4.48 (2.4–8.31)	<0.01
Neoplasm, n/N (%)	16/66 (24)	17/194 (9)	3.18 (1.49–6.78)	<0.01
Gastrointestinal, n/N (%)	15/66 (23)	19/194 (10)	2.71 (1.29–5.71)	0.01
Hepatic, n/N (%)	18/66 (27)	41/194 (21)	1.40 (0.74–2.66)	0.31
Others, n/N (%)	25/66 (38)	49/194 (25)	1.8 (1.00–3.27)	0.05
Functional status				
SPPB impaired, n/N (%)	31/66 (47)	1/36 (3)	30 (3.95–229)	<0.01
Barthel impaired, n/N (%)	1/66 (2)	0/36 (0)	3.02 (0.12–75.4)	1.00
Lawton & Brody impaired, n/N (%)	16/66 (24)	0/36 (0)	27.7 (1.66–464)	<0.01
Geriatric syndromes				
Sleep disturbance, n/N (%)	16/66 (24)	4/194 (2)	15.2 (4.87–47.48)	<0.01
Malnutrition risk, n/N (%)	34/66 (52)	37/194 (19)	4.5 (2.47–8.22)	<0.01
Visual impairment, n/N (%)	30/66 (46)	16/194 (8)	9.27 (4.58–18.76)	<0.01
Hearing impairment, n/N (%)	36/66 (55)	56/191 (29)	2.89 (1.63–5.15)	<0.01
Sexual dysfunction, n/N (%)	53/66 (80)	79/156 (51)	3.97 (2.01–7.87)	<0.01
Neurocognitive & Mental Health				
Cognitive complaint, n/N (%)	27/66 (41)	13/194 (7)	9.64 (4.57–20.34)	<0.01
Cognitive performance, n/N (%)	25/53 (47)	17/194 (9)	9.30 (4.46–19.36)	<0.01
Depressive symptoms, n/N (%)	44/64 (69)	45/185 (24)	6.84 (3.66–12.80)	<0.01
Quality of life (by a self-developed instrument) & Social support (by OARS)				
Low quality of life (QoL), n/N (%)	25/60 (42)	12/163 (7)	8.99 (4.12–19.61)	<0.01
Good social support, n/N (%)	12/21 (57)	41/45 (91)	0.13 (0.03–0.50)	0.02
Mild deterioration, n/N (%)	6/21 (29)	4/45 (9)	4.1 (1.01–16.57)	0.06
Severe deterioration, n/N (%)	2/21 (10)	0/45 (0)	11.7 (0.53–254.46)	0.09

Data are presented as median (interquartile range [IQR]) or number and proportion (%). Categorical variables were compared using Chi-square or Fisher’s exact test; continuous variables were analyzed using the Mann–Whitney U test. Statistical significance was set at *p* < 0.05. Odds ratios (OR) with 95% confidence intervals (CI) were calculated using exact methods. Tests performed using 2 × 2 tables (presence vs. absence) by gender, *p*-values indicate statistical significance at alpha = 0.05 without adjustment for multiple comparisons. Frailty was defined using Fried criteria.

**Table 3:** Gender-based differences among frail PWH (WWH vs. MWH).

Variable	Frail WWH (n = 24)	Frail MWW (n = 41)	OR (95% CI)	*p*-Value
Demographics				
≥60 years, n/N (%)	9/24 (37)	24/41 (59)	0.42 (0.15–1.18)	0.13
≥70 years, n/N (%)	6/24 (25)	13/41 (32)	0.72 (0.23–2.23)	0.78
HIV-Related data				
CD4+ ≤200 count cells/mm^3^, n (%)	3/24 (13)	2/41 (5)	2.77 (0.41–18.7)	0.35
Comorbidity burden				
≥3 comorbidities, n/N (%)	22/24 (92)	33/41 (81)	2.70 (0.47–15.6)	0.30
Comorbidity domains				
Endocrine, n/N (%)	15/24 (63)	24/41 (59)	1.20 (0.43–3.37)	0.80
Cardiovascular, n/N (%)	10/24 (42)	20/41 (49)	0.75 (0.27–2.07)	0.62
Renal, n/N (%)	6/24 (25)	12/41 (29)	0.80 (0.25–2.60)	0.78
Musculoskeletal, n/N (%)	20/24 (83)	17/41 (41)	6.88 (2.04–23.2)	<0.01
Neurological, n/N (%)	6/24 (25)	12/41 (29)	0.81 (0.25–2.64)	0.78
Respiratory, n/N (%)	9/24 (38)	13/41 (32)	1.29 (0.45–3.70)	0.79
Psychiatric, n/N (%)	15/24 (63)	16/41 (39)	2.69 (0.92–7.85)	0.07
Neoplasm, n/N (%)	9/24 (38)	7/41 (17)	2.93 (0.86–9.97)	0.08
Gastrointestinal, n/N (%)	4/24 (17)	11/41 (27)	0.56 (0.15–2.12)	0.54
Hepatic, n/N (%)	7/24 (29)	11/41 (27)	1.11 (0.36–3.44)	1.00
Others, n/N (%)	11/24 (46)	14/41 (34)	1.65 (0.57–4.77)	0.43
Functional status				
SPPB impaired, n/N (%)	12/24 (50)	20/39 (51)	0.97 (0.36–2.61)	1.00
Barthel impaired, n/N (%)	1/24 (4)	0/41 (0)	0.47 (0.06–1.13)	0.36
Lawton & Brody impaired, n/N (%)	3/22 (14)	13/41 (32)	0.35 (0.09–1.42)	0.43
Geriatric syndromes				
Sleep disturbance, n/N (%)	7/24 (29)	9/41 (22)	1.14 (0.38–3.44)	0.56
Malnutrition risk, n/N (%)	11/24 (46)	23/41 (56)	0.67 (0.25–1.79)	0.45
Visual impairment, n/N (%)	5/18 (28)	21/41 (51)	0.38 (0.15–0.95)	0.02
Hearing impairment, n/N (%)	12/24 (50)	24/41 (59)	0.69 (0.25–1.88)	0.61
Sexual dysfunction, n/N (%)	20/24 (83)	34/41 (83)	1.00 (0.27–3.70)	1.00
Neurocognitive & Mental Health				
Cognitive complaint, n/N (%)	15/24 (63)	12/41 (29)	4.03 (1.4–12)	0.01
Cognitive performance, n/N (%)	9/20 (45)	16/32 (50)	0.82 (0.27–2.51)	0.78
Depressive symptoms, n/N (%)	16/23 (70)	28/40 (70)	0.98 (0.32–3)	1.00
Quality of life (by a self-developed instrument) & Social support (by OARS)				
Low quality of life (QoL), n/N (%)	11/24 (46)	14/34 (41)	1.21 (0.42–3.47)	0.79
Good social support, n/N (%)	3/7 (43)	9/13 (69)	0.33 (0.05–2)	0.35
Mild deterioration, n/N (%)	1/7 (14)	1/13 (8)	2 (0.11–38)	1.00
Severe deterioration, n/N (%)	3/7 (43)	3/13 (23)	3 (0.35–18)	0.61

Data are presented as median (interquartile range [IQR]) or number and proportion (%). Categorical variables were compared using Chi-square or Fisher’s exact test; continuous variables were analyzed using the Mann–Whitney U test. Statistical significance was set at *p* < 0.05. Odds ratios (OR) with 95% confidence intervals (CI) were calculated using exact methods. Tests performed using 2 × 2 tables (presence vs. absence) by gender, *p*-values indicate statistical significance at alpha = 0.05 without adjustment for multiple comparisons.

**Table 4:** Gender-stratified multivariable analysis of frailty in people with HIV.

Variable	WWH (Adjusted OR, 95% CI)	*p*-Value	MWH (Adjusted OR, 95% CI)	*p*-Value	*p* for Interaction
Age ≥ 70 years	1.72 (0.91–3.24)	0.09	2.94 (1.85–4.68)	<0.01	**0.03**
Musculoskeletal disease	3.85 (1.72–8.61)	<0.01	2.21 (1.34–3.66)	<0.01	0.12
Cognitive complaint	1.88 (0.94–3.74)	0.07	1.29 (0.82–2.04)	0.27	0.17
Cognitive impairment	3.21 (1.45–7.10)	<0.01	2.54 (1.48–4.35)	<0.01	0.28
Depressive symptoms	2.67 (1.28–5.54)	<0.01	1.41 (0.85–2.32)	0.18	0.04
Malnutrition	2.14 (1.02–4.51)	0.04	2.36 (1.33–4.18)	<0.01	0.67
Sleep disturbance	1.96 (0.89–4.30)	0.09	1.22 (0.74–2.01)	0.42	0.21

Data are presented as adjusted odds ratios (aOR) with 95% confidence intervals (CI). Models were stratified by gender. Interaction *p*-values reflect sex differences in associations. The model includes all variables listed. Frailty was defined according to Fried criteria. Covariates were selected a priori based on clinical relevance.

## Data Availability

All data generated or analyzed during this study are included in this published article.

## Abbreviations

The following abbreviations are used in this manuscript:

PWH	People with HIV
WWH	Women with HIV
MWH	Men with HIV
ART	Antiretroviral therapy
IQR	Interquartile range
SPPB	Short Physical Performance Battery
VAS	Visual Analog Scale for pain
MNA	Mini Nutritional Assessment
EACS	European AIDS Clinical Society cognitive screening questions
NEU	Neurocognitive Screening
GDS	Geriatric Depression Scale
QoL	Quality of life
OARS	Older Americans Resources and Services
OR	Odds ratio
CI	Confidence interval
